# Sustained impact of energy-dense TV and online food advertising on children’s dietary intake: a within-subject, randomised, crossover, counter-balanced trial

**DOI:** 10.1186/s12966-018-0672-6

**Published:** 2018-04-12

**Authors:** Jennifer Norman, Bridget Kelly, Anne-T McMahon, Emma Boyland, Louise A. Baur, Kathy Chapman, Lesley King, Clare Hughes, Adrian Bauman

**Affiliations:** 1 Early Start, School of Health and Society, Faculty of Social Sciences, University of Wollongong, Wollongong, NSW 2522 Australia; 2 School of Health and Society, Faculty of Social Sciences, University of Wollongong, Wollongong, NSW 2522 Australia; 3 Appetite & Obesity Research Group, Department of Psychological Sciences, University of Liverpool, Liverpool, L69 7ZA UK; 4 Prevention Research Collaboration, School of Public Health, University of Sydney, Sydney, NSW 2006 Australia; 5 Cancer Council NSW, 153, Dowling Street, Woolloomooloo, NSW 2011 Australia

**Keywords:** Food advertising, Advergame, Children, Food intake, Dietary intake, Childhood overweight, Childhood obesity

## Abstract

**Background:**

Policies restricting children’s exposure to unhealthy food marketing have been impeded by the lack of evidence showing a direct link between food advertising exposure and children’s energy intake and body weight. Food advertising exposure increases children’s immediate food consumption, but whether this increased intake is compensated for at later eating occasions is not known; consequently the sustained effect on diets remains unclear.

**Methods:**

We conducted a within-subject, randomised, crossover, counterbalanced study across four, six-day holiday camps in New South Wales, Australia between April 2016 and January 2017. Children (7–12 years, *n* = 160) were recruited via local schools, email networks and social media. Two gender- and age-balanced groups were formed for each camp (*n* = 20), randomised to either a multiple- or single- media condition and exposed to food and non-food advertising in an online game and/or a television cartoon. Children’s food consumption (kilojoules) was measured at a snack immediately after exposure and then at lunch later in the day. Linear mixed models were conducted to examine relationships between food advertising exposure and dietary intake, taking into account gender, age and weight status.

**Results:**

All children in the multiple-media condition ate more at a snack after exposure to food advertising compared with non-food advertising; this was not compensated for at lunch, leading to additional daily food intake of 194 kJ (95% CI 80–308, *p* = 0.001, d = 0.2). Exposure to multiple-media food advertising compared with a single-media source increased the effect on snack intake by a difference of 182 kJ (95% CI 46–317, *p* = 0.009, d = 0.4). Food advertising had an increased effect among children with heavier weight status in both media groups.

**Conclusion:**

Online (‘advergame’) advertising combined with TV advertising exerted a stronger influence on children’s food consumption than TV advertising alone. The lack of compensation at lunch for children’s increased snack intake after food advertising exposure suggests that unhealthy food advertising exposure contributes to a positive energy-gap, which could cumulatively lead to the development of overweight.

**Trial registration:**

Australian New Zealand Clinical Trials Registry, number ACTRN12617001230347 (Retrospectively registered).

## Background

Overweight is, arguably, the natural response to our food environment [1] which is dominated by low-cost, ultra-processed, energy-dense, highly palatable food products [2]. Food marketing most commonly promotes these high fat, high salt and high sugar foods [3]. Worldwide, television is still the main platform for food advertising [3, 4], however, the proliferation of digital technologies, including the Internet and mobile devices, has seen an increasing prevalence of food advertising on ‘new media’ [5]. In recent years, advergames have been introduced as an online marketing tool, where the brand and/or product is a prominent feature [6]. This high prevalence of unhealthy food promotion propagates societal norms where advertised high energy and low nutrient dense foods are acceptable and desirable [6]. Advertisements also serve as conditioned stimuli, which stimulate food cravings and cue consumption [7].

Restricting children’s food marketing exposure has been identified as an international policy priority for the prevention of childhood overweight and obesity [8, 9]. However, few countries have enforced statutory regulations and, globally, major regulatory reform essentially remains un-implemented, with most countries relying on industry-led pledges for responsible advertising [10]. Research evidence indicates that these industry pledges have not been effective in reducing children’s exposures to unhealthy food marketing [10, 11]. As such, children continue to be exposed to high levels of unhealthy food marketing across a wide variety of media and settings [11], to promotions they find highly appealing and engaging [3]. A growing body of evidence indicates that food marketing affects children’s food attitudes, preferences and consumption [3, 12], most likely through a logical, cumulative sequence of cognitive and behavioural responses [13]. A key issue impeding policy change, however, is the shortage of evidence showing a direct link between food marketing and children’s energy intake and the sustained effect of exposures on children’s body weight [14]. Brief exposure to food advertising on TV or Internet advergames has an immediate direct effect on children’s food consumption, significantly increasing their intake of snack foods [15], but whether or not this increased energy intake is compensated for at later eating occasions is not known. Many of these single exposure experimental studies have been conducted in laboratory settings and have not accounted for the cumulative effects of media exposures or the impact of repeated exposures across multiple media.

Economic modelling suggests that limiting food marketing to children would be one of the most cost-effective population-based strategies to reduce the prevalence of childhood obesity, resulting in both children’s health gains and health-service savings [16]. Data that were used to calculate these cost-benefits are now over three decades old, being derived from the only longer-term experimental study in this field, conducted at a children’s holiday camp in Canada in 1982 [17]. The advertising landscape of 2018 is vastly different [5] and up-to-date data is needed for contemporary economic modelling studies. Conducting longer-term experimental studies in this field, however, is methodologically challenging and resource intensive and, as such, research of this nature is limited [13].

This study aimed to document children’s dietary intake over a period of six days during their time at a holiday camp, following exposure to food and non-food advertising from online (advergames) and/or TV media platforms. There were three main objectives for this study. First, we tested the hypothesis that children would eat more at a snack after food advertising exposure compared with non-food advertising. Secondly, we hypothesised that exposure to food advertising across multiple media would have an increased effect on children’s immediate snack intake compared with those only exposed to food advertising from a single media source. Thirdly, this study measured if any increased energy consumed as a result of exposure to food advertising was compensated for by children consuming less energy at the later lunchtime eating occasion, and hence identified whether food advertising exposure resulted in a positive energy balance during their time at camp.

## Methods

### Study design, participants and materials

The study took place across four, six-day school holiday camps, from 8 am to 1.30 pm each day, between April 2016 and January 2017, at a single location in New South Wales (NSW), Australia. We partnered with the University of Wollongong Children’s Sports Holiday Camp and the Early Start research centre. Early Start is a child-focused research facility incorporating a large commercial kitchen, dining area and community engagement rooms. Both the camp and research centre are located on the same campus, within five minutes’ walk of each other.

Participant recruitment took place in the month preceding each holiday camp period, in March, June, July and December 2016. A total of 160 children, (78 female, 82 male), aged 7–12 years (9.3 ± 1.6 (mean ± SD), were recruited into the camp (*n* = 40/camp) via local schools, community and university networks and social media. Children were deemed eligible if they were able to attend the camp on all days; did not report having any food allergies or intolerances or medical conditions affecting what they could eat; had no dietary restrictions or dislike of the study foods; and were able to sit still and focus on a task for at least 15 min. Incentives for participants included payment for their holiday camp fees and the opportunity to enter a draw to win an iPad at the end of each camp. Children were only permitted to participate once in the study, attending only one of the holiday camps during the data collection period. The study protocol was approved by the University of Wollongong Human Research Ethics Committee and can be accessed at http://www.ANZCTR.org.au/ACTRN12617001230347.aspx. Informed written consent from parents was obtained for all participants.

**Fig. 1:**
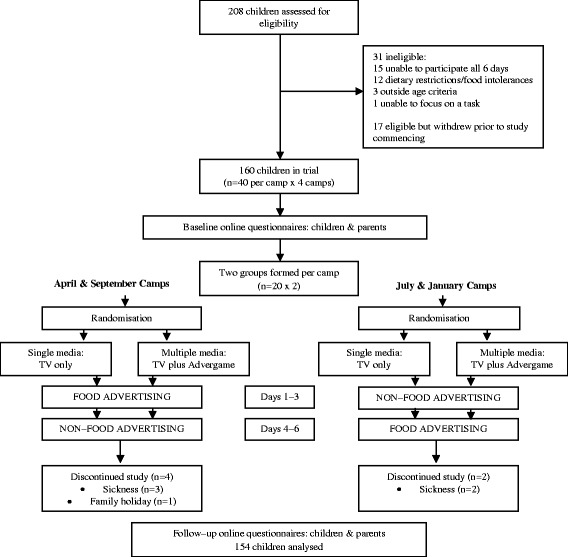
Consort flow diagram and study design

The study was a within-subject, randomised, crossover, counterbalanced trial with two media condition arms: multiple media (TV plus advergame) or single media (TV only) (Fig. 1). Each media arm had two conditions: control (an advergame featuring a non-food brand and/or exposure to ten non-food TV advertisements) and experimental (an advergame featuring an unhealthy food brand and/or exposure to ten unhealthy TV food advertisements). Food products in the experimental condition were classed as high in fat, salt and/or sugar in accordance with the nutrient profiling criteria developed by Food Standards Australia New Zealand [18]. Non-food advertisements were selected on the basis that they used persuasive techniques such as fun, action and promotional characters; themes that are commonly used in food advertisements to appeal to children [19]. (A list of the advertised products that were used is detailed in Table 1.) The TV advertisements (approximately 30 s each) were embedded within a ten minute age-appropriate, gender-neutral cartoon and shown each day. There were no references to, or depictions of, foods or eating in any of the cartoons screened. In order to isolate the effects of the study advertising exposure, the branded products selected for the experimental condition were real products available in other countries but not available for sale in Australian supermarkets or advertised on commercial TV within Australia. Two groups of 20 children were formed for each camp, with an approximate even spread of gender and age between groups. A simple, manual randomisation method was used, with the first group drawn out of a hat allocated to the single media intervention. This was conducted by an independent researcher not associated with the study. Children undertook both advertising conditions in each intervention arm, with the order of advertising condition counterbalanced across holiday camps. The study protocol, including menu items, was finalised following a pilot study with 30 children in January 2016.

**Table 1:** Branded products featured in advergames and TV advertisements in each condition

Experimental (Food Advertisement Condition)	Control (Non-Food Advertisement Condition)
Advergame:	
Nestle Kokokrunch cereal	Lego
TV advertisements:	
McVities Delichoc biscuit	The North Face sportswear
Burger King Meal Deal	Vodafone Ireland
Taco Bell Smart Phone App	Speedo swimwear
McCoys crisps	Visit England
Nestle Kokokrunch cereal	O2 telecommunications
Hersheys chocolate spread	British Airways
McVities BN biscuit	Bloomingdales
Walkers Mixups crisps	Mini
Hula Hoops savoury snacks	Disney Cruise Line
Maynards Discovery Patch confectionery	Petplan insurance

An online brand recognition tool was designed to assess children’s recollection of the advertised food brands, both pre and post study. Children were asked: a) if they recognised 20 different photographs of both food and non-food logos, and b) to describe the product to which the logo related. This tool was based on a validated food brand recognition instrument [20].

Children’s height and weight were measured on Day 1 of the study. Children’s body mass index (BMI) was calculated and these values were used to classify children into underweight, normal weight, overweight or obese categories using international standardised cut-points [21].

Children reported how hungry they felt prior to morning tea and lunch each day using a validated picture-rating scale; with the anchors “I am really hungry” and “I am not hungry at all” [22].

### Procedure

Each morning, children arrived fasting at the research centre at 8 am, where they were served breakfast. The dining space was set up with 40 individual trays and, each day, children were offered a selection of portioned breakfast cereals, fruit, toast or pikelets, spreads and water to drink. The children were told that they could eat as much or as little as they liked, and were given more of each food item as requested. All additional food items were offered in pre-portioned amounts that were the same size for all children. Children were allocated a unique identifying number which was placed on their meal tray at each eating occasion and used to track how much each child ate daily. After 30 min, the children left to participate in the nearby holiday camp activities. Camp leaders ensured that the physical intensity of camp activities was similar each session and day of the sports camp. Children’s breakfast intake was quantified by assessing whether children had eaten all, more than half, half, less than half or none of each food item. Visual estimation methods have been demonstrated to be a valid and highly reliable method for estimating children’s food and beverage consumption of pre-portioned food items [23]. Kilojoules (kJ) were estimated from the proportion of each standardised food item consumed, and children’s mean breakfast intakes for the three days of food and three days of non-food advertising were estimated.

At mid-morning children were directed to their intervention rooms in the research centre with each room supervised by two research assistants. The rooms were bright and colourful with a carpeted floor and a large, wall mounted TV screen. Children in both intervention rooms were asked to take a seat on the floor and to report how hungry they felt by filling out the picture rating scale [22]. Children were then told that they would be watching a cartoon and some advertisements. Once the cartoon had finished, iPads were distributed to the children in the multiple media condition and, after an explanation on how to play, they played an advergame for 5 min. In both rooms, after media exposure was complete, individual trays with six small bowls of snack foods were given out. Bowls contained 50 g of each of the following foods: high fat savoury (crinkle-cut crisps, plain potato crisps or chicken-flavoured crackers); low fat savoury (pretzels, plain crackers or rice crackers); high fat sweet (milk chocolate, chocolate-covered biscuits or sugar-coated chocolate confectionery); low fat sweet (assorted jelly lollies); fruit (green and black grapes or peeled mandarin segments); and vegetable (carrot sticks). These selections were in line with previous study designs [24]. Snack items offered on Day One were matched with those offered on Day Four, Day Two with Day Five and Day Three with Day Six. None of the brands offered to children to eat were featured in any of the advertisements. Children were asked to wait until everyone had their trays before they could start eating. They were, again, told that they could eat as little or as much as they liked, and if they would like some more they should put their hand up and they would be given more of the requested food. This was the only time either food or eating was referred to by the research assistants. All additional foods were pre-weighed in advance of the morning tea period. Advertisements were not discussed with the children. Eating time was limited to 15 min after which children were instructed to leave the room. Afterwards children left to take part in further holiday camp activities and the remaining food on each child’s tray was weighed and recorded.

Lunch was served at 13.00 each day back in the research centre. Once again 40 individual trays were set up with pre-weighed food items. There was a different menu for Days One to Three which was repeated on Days Four to Six. Lunch items included fruit, vegetables, yoghurt and healthier versions of fast food, e.g. low fat beef burger; oven baked chicken pieces and chips. Each day prior to lunch, children completed the picture rating scale to report how hungry they felt [22]. As with the previous meal occasions, children were told that they could eat as little or as much as they liked and if they would like some more to ask and they would be given more of the requested food. Again, all additional food items were pre-weighed prior to the lunchtime period. Parents arrived to pick children up at 13.30. Once children had left, all remaining individual food items were weighed and recorded. We did not collect dietary data from children once they left camp for the day.

Children’s morning snack and lunch intakes were converted from gram amounts to kJ using FoodWorks 8 nutrient analysis software.

Children completed the online brand recognition questionnaires at home prior to the study commencing and in the week following the study’s completion and a brand recognition score was calculated. Parents reported their household weekly income via an online questionnaire at the end of the study.

### Outcomes

There were two primary outcomes: firstly whether there was an increase in snack intake (kJ) after food advertising exposure compared with non-food advertising exposure and secondly, if any increased intake was compensated for at the lunchtime meal. The secondary outcome was whether there was an increased effect on energy intake (kJ) from exposure to food advertising over multiple media compared with a single media source.

### Statistical analysis

The sample size, with sufficient statistical power (80%) to assess the first primary outcome, with a significance of 0.05, was estimated from published data from a similar, short-term advertising exposure feeding trial in the UK using the differences in kJ reported between conditions [25].

Each child’s mean snack and lunch intakes for the three days of food advertising exposure and three days of non-food advertising exposure were calculated. All intake data met normality assumptions. Analysis of the primary and secondary outcomes was conducted using linear mixed models, adjusting for the clustered nature of the data (i.e. camp identifier was included as a random intercept in the models). The linear mixed models were used to examine the differences in snack intake (kJ) between the two media groups and the differences in the snack and lunch intakes (kJ) between advertising conditions within each group. Any influence of the impact of age (months), gender, weight status (BMI z-score), children’s baseline brand recognition score, household weekly income or hunger on snack intake was investigated by adding these variables as covariates to the model. All analyses used a significance level of 0.05. All analyses were completed using the Statistical Package for the Social Sciences statistical software package, version 23 (SPSS Inc., Chicago, IL, USA).

## Results

Of the 160 children enrolled in the study, six did not complete all six days of the camp, so their data were not included in the final analysis (Fig. 1). Table 2 depicts the completing participants’ characteristics across the two media condition groups. The proportion of children with overweight or obesity in our study (16%) was lower than the NSW state average (23%) [26]. The median household income of all families in the study was between $2000–2499 per week which was substantially higher than the NSW median household income of $800–999 per week [27].

**Table 2:** Participant Characteristics by Media Condition Group

	TV only (*n* = 76)	TV plus advergame (*n* = 78)	All (*n* = 154)
Gender, n (%)			
Male	37 (48.7)	40 (51.3)	77 (50.0)
Female	39 (51.3)	38 (48.7)	77 (50.0)
Age, mean ± SD (range), y	9.6 ± 1.5 (7.0–12.3)	9.1 ± 1.8 (6.5–12.9)	9.3 ± 1.6 (6.5–12.9)
BMI for age, n (%) [21]			
Underweight	4 (5.2)	1 (1.3)	5 (3.3)
Normal weight	61 (80.3)	63 (80.8)	124 (80.5)
Overweight	10 (13.2)	9 (11.5)	19 (12.3)
Obesity	1 (1.3)	5 (6.4)	6 (3.9)
Median household weekly income ($)	1500–1999*	2000–2499**	2000–2499

*5% did not answer; **14% did not answer

As a whole group (*n* = 154), children’s estimated mean kJ intake at breakfast on the food advertising days (1350 ± 500 kJ) was similar to intakes on non-food advertising days (1300 ± 500 kJ) (*p* = 0.079).

A significant main effect for media condition was found. Children in the TV plus advergame group (*n* = 78) ate more at the snack after food advertising compared with non-food advertising than the TV only group (*n* = 76) (additional 182 kJ (95% CI 46 to 317)) (*p* = 0.009, d = 0.4) (Table 3). Consequently, data were analysed separately by media condition. Age, gender, brand recognition score at baseline and household weekly income had no significant main effect or interaction and were removed from further analyses.

**Table 3:** The effects of advertisement condition on the kJ intake in all children and by weight status across both media conditions

	Difference in means between food and non-food ads SNACK (kJ)	Difference in means between food and non-food ads LUNCH (kJ)	Additional energy intake per day at the holiday camp after food advertising SNACK PLUS LUNCH (kJ)
Whole group			
All children (*n* = 154)	111 (434) *p* = 0.002, d = 0.2	41 (397)	152 (556) *p* = 0.001, d = 0.2
Under−/normal weight (*n* = 129)	90 (414) *p* = 0.015, d = 0.1	1 (388)	91 (521) *p* = 0.05, d = 0.1
Overweight/obesity (*n* = 25)	221 (521) *p* = 0.045, d = 0.3	246 (389) *p* = 0.004, d = 0.4	467 (631) *p* = 0.001, d = 0.4
TV only			
All children (*n* = 76)	19 (460)	89 (413)	108 (603)
Under−/normal weight (*n* = 65)	4 (427)	29 (378)	33 (522)
Overweight/obesity (*n* = 11)	113 (647)	441 (456) *p* = 0.009, d = 1.1	554 (858) *p* = 0.058, d = 0.6
TV plus advergame			
All children (*n* = 78)	201 (388) *p* < 0.0001, d = 0.3	–6 (377)	194 (388) *p* = 0.001, d = 0.2
Under−/normal weight (*n* = 64)	178 (385) *p* < 0.0001, d = 0.3	−28 (398)	150 (518) *p* = 0.024, d = 0.2
Overweight/obesity (*n* = 14)	305 (402) *p* = 0.014, d = 0.3	93 (248)	398 (398) *p* = 0.002, d = 0.3

Mean (kJ) (SD). All *p* values are two tailed. d = effect size, Cohen’s d

Children’s reported hunger prior to advertising exposure was related to their snack intake in both the food advertising and non-food advertising conditions (*p* = 0.000). The difference in snack intake between food and non-food advertising exposures, however, remained significant after controlling for the difference in hunger between the advertising conditions (*p* = 0.008). In the TV only condition, BMI z-score was related to children’s snack intake after food advertising (*p* = 0.003) and non-food advertising (*p* = 0.038).

In the TV plus advergame group (*n* = 78), mean kJ intake was 201 kJ higher at the snack after food advertising exposure (2168 ± 787 kJ) compared with non-food advertising (1968 ± 698 kJ) (*p* < 0.0001, d = 0.3). This difference remained significant after controlling for hunger (*p* < 0.0001). Within this media group, children with overweight or obesity (*n* = 14) ate an additional 305 kJ (95% CI 73 to 538) more after food advertising exposure (*p* = 0.014, d = 0.3) compared with children with under−/normal weight (*n* = 64) who ate 178 kJ (95% CI 82 to 274) more (*p* < 0.0001, d = 0.3). In the TV only group, children with under−/normal weight (*n* = 65) ate comparable amounts after both food (1933 ± 619 kJ) and non-food (1929 ± 678 kJ) advertising exposures (*p* = 0.947). Likewise, within this group, children with overweight or obesity (*n* = 11) also ate similar amounts in both the food (2220 ± 711 kJ) and non-food advertising (2107 ± 621 kJ) conditions (*p* = 0.576).

**Fig. 2:**
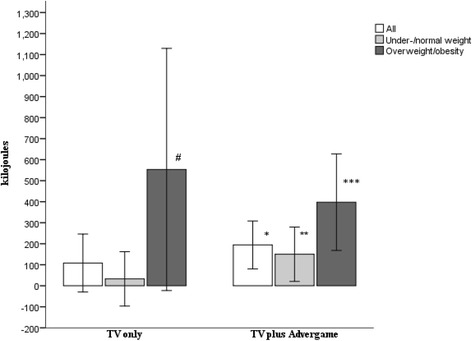
Mean daily additional kJ (95% CI) consumed at the camp after exposure to food advertising by children with under−/normal weight and overweight or obesity within the two media conditions. * Significant increase in total kJ consumed after food advertising compared with non-food advertising (*p* = 0.001). ** Significant increase in total kJ consumed after food advertising compared with non-food advertising (*p* = 0.024). *** Significant increase in total kJ consumed after food advertising compared with non-food advertising (*p* = 0.002). # Non-significant increase in total kJ consumed after food advertising compared with non-food advertising (*p* = 0.058)

Children in the TV plus advergame group did not compensate for their increased snack intake after food advertising at their lunchtime meal. Children in this group consumed an additional 194 kJ (95% CI 80 to 308) daily at camp (snack + lunch) (*p* = 0.001) after food advertising exposure compared with non-food advertising exposure (Fig. 2, Table 3). The effect of the food advertising appeared to be greater among children with heavier weight status. Children with overweight and obesity in the TV plus advergame group consumed an extra 398 kJ (95% CI 168 to 627) daily at camp (*p* = 0.002) compared with children with under−/normal weight who consumed an extra 150 kJ (95% CI 21 to 279) daily (*p* = 0.024) (Fig. 2). In the TV only group, children with overweight or obesity ate an extra 441 kJ (95% CI 135 to 747) at lunch on food advertising days (*p* = 0.009); this led to an additional 553 kJ (95% CI –23 to 1129) being consumed on food advertising days at camp compared with non-food advertising days (*p* = 0.058).

## Discussion

### Principal findings of the study

Exposure to food advertising on TV and the advergame led to significant increases in children’s daily energy intake during their time at camp (194 kJ). This response was magnified among children with overweight and obesity (*n* = 14); consuming over double the additional daily kJ at the camp (398 kJ) than children with under−/normal weight (*n* = 64, 150 kJ). This exaggerated response was also seen among children of a heavier weight status in the TV only group, with children (*n* = 11) consuming an extra 553 kJ (*p* = 0.058) at the camp on the food advertising days. Given that cohort studies suggest that a positive energy-gap of only 200–300 kJ a day may be all that is required for the development of overweight in children [28, 29], these data raise legitimate concerns about the direct influence that food advertising over long-term exposures may exert on children’s weight.

### Interpretation

Whilst not consistently seen across previous studies, children with a heavier weight status have been often shown to have heightened food intake responses to food advertising on TV [24, 30] and branded food packages [31]. In a UK study, children with overweight (*n* = 15) increased their intake by 1280 kJ after TV food advertising compared with control, and children with obesity (*n* = 11) by 1970 kJ (*p* < 0.001), indicating they were highly influenced by external food cues [24]. Indeed, children with overweight or obesity, when tested with a ‘stop signal’ task, have been shown to have less inhibitory control in response to food stimuli than children with normal weight [32]. The pre-frontal cortex is an area of the brain involved in cognitive control, including impulse inhibition, and making decisions in response to stimuli in the environment [33]. Neural imaging studies indicate a lack of activation of this region in children in response to food cues, in contrast to adults where activation is apparent [33]. In response to fast food logos, children with obesity have been shown to have lower activation in the pre-frontal cortex compared with children with normal weight [34]. These data suggest that a lack of impulsivity inhibition in children, in response to food cues, could make them less able to control eating impulses and, thus, more susceptible to tempting food stimuli such as those found in food marketing. Additionally, it would also appear that children with a heavier weight status have a particular vulnerability to branding and food advertising effects.

Children within the TV plus advergame group ate significantly more snack foods after food advertising (201 kJ) compared with non-food advertising, whilst children in the TV only group ate similar amounts at the snack after both the food and non-food advertisements. There was an increased effect seen among children with a heavier weight status in both media groups but at different meal occasions. In the TV plus advergame group children with overweight or obesity (*n* = 14) increased their snack intake by a significant 305 kJ after food advertising exposure. In the TV only group (*n* = 11), although no increased effect was observed at snack-time, children with overweight and obesity ate an additional 441 kJ at lunchtime on food advertising days compared with non-food advertising days. The advertised foods were not offered to the children, indicating these effects were not brand or product specific but transferred to the foods that were on offer. Significant effects also remained after controlling for hunger. The lack of an advergame only group meant that we were unable to isolate whether the significant main effect for media condition that we observed was due to the double exposure of TV and the advergame or to the nature of the game itself. The effects of playing an advergame only on snack intake have been previously demonstrated, with children (8–10 years) eating more (286 kJ, *p* < 0.001) after playing a five minute game promoting energy-dense foods (*n* = 69) compared with a non-food game (*n* = 65); although this study did not differentiate between children with different weight statuses [35]. Advergames are designed to be fun and engaging, with brand immersion as the primary objective [36]. Given the consumption responses of children within this group, it would appear children have responded to the food cues prominent within the games.

Our findings suggest insights into child obesity policy at both upstream and mid-stream levels. From a primary prevention perspective, our findings clearly highlight the need for regulatory intervention to restrict children’s exposure to unhealthy food promotions, on TV and across different media platforms, and particularly online media. The recent rules introduced by the UK’s media self-regulatory body, the Committee of Advertising Practice, restricting the advertising of foods high in fat, salt or sugar in children’s non-broadcast media, are a step in the right direction in this regard [37]. From a weight management perspective, the increased vulnerability to food cues we observed among children with a heavier weight status supports the inclusion of interventions which aim to diminish sensitivity to these stimuli in children’s weight loss education programs [7, 38, 39]. Although in their early infancy as an approach for children’s weight management, Cue Exposure Treatment programs hold promise in supporting children to resist the abundance of food cues in today’s environment [7, 38, 39]. The basic premise of these interventions is to present children with repeated, non-reinforced exposures to highly palatable foods with the aim of weakening the child’s conditioned response, that is, the desire to eat [38, 39].

### Strengths and limitations

A key strength of this study was that we created a natural environment within which to collect the data, and in which the children were comfortable and relaxed. The recreation centre and the dining area would not be dissimilar to the facilities that children would experience when attending school camps during term time. That the children enjoyed taking part in the study and camp is strongly supported by the overwhelmingly positive feedback we received from the parents in their follow-up questionnaire and the low study attrition.

Some limitations in study design point to why significant differences in snack intake between food and non-food advertising conditions in the TV only group were not seen. Unlike previous studies, which used TV food advertisements aired within their study’s countries [24, 30, 40], we elected to use unfamiliar brands. This was to isolate the effect of advertising exposure, without influence from pre-formed brand attitudes or associations. There is strong evidence that repetitive exposure to advertising enhances evaluation of that stimuli [41, 42], with maximum attitude and affect reached at approximately ten advertising exposures [43]. It is possible that exposure to novel brands and, consequently lesser opportunity to form positive affect with that brand or product, may not have cued children’s consumption responses to the extent that has previously been observed where food brands were familiar [24, 30, 40], thus resulting in a null effect. The increased effect of prior advertising exposure on children’s food intake exposure via advergames has previously been demonstrated. In an earlier study from the USA, children who had previous food branded game play experience consumed 577 kJ more from unhealthy snack foods than children who played healthy or non-food advergames in the study [44].

The time of day we conducted our study could also have influenced children’s consumption responses. Evidence suggests that self-regulatory capacity lessens across time as the cognitive resources needed to exert inhibition and executive control become depleted over the course of the day [45]. We measured children’s snack intake in the morning, when children’s ability to self-regulate is, arguably, at its height [45]. In contrast, previous studies, where significant effects of TV food advertising on children’s food intake were seen, and which reported time of day, were conducted in the afternoon [17, 40].

A further limitation may have been that children were given a 15 min snack eating time, which was not disclosed to them. In two previous studies, where significant effects on snack food intake were seen subsequent to TV food advertising exposure, children (*n* = 42–59, aged 9–11 years) were given unlimited time to eat during the experimental eating period [24, 30]. In our study, a number of children had asked for more high fat sweet or low fat sweet items and had not eaten all that they had requested. We surmise that having asked for more of these food items that children had the intention of eating them and, hence, would have finished eating the sweets if they had had sufficient time. Indeed, the most proximal determinant of actual behaviour is the intention to carry out that behavior, according to the Theory of Planned Behaviour [46]. Food enjoyment is inversely correlated with satiety responsiveness and positively correlated with food responsiveness and desire to eat [47]. The portion size of the extra food items was only 25 g so coupled with the low satiability of energy-dense, sweet and high fat foods [48, 49], and with children’s predisposition for these highly palatable foods [50], this is a reasonable expectation. Analyses of mean snack food intakes allowing for children to finish the extras that they had asked for, suggests that food advertising would have had a significant increased effect on children’s snack intake in the TV only condition (data not shown).

Furthermore, we were under-represented in the proportion of children with overweight or obesity in our study compared with the state population [26] and, hence, it would appear, under-powered to detect a mean difference in snack intake among this subgroup of children in the TV only group. A power calculation indicates that we would have needed a sample of 60 children with overweight or obesity to detect a mean difference of the magnitude that we observed (113 kJ), with a significance level of 0.05.

In line with previous studies [51, 52], we asked children to report how hungry they were feeling prior to the snack period, in order to control for hunger in our analyses. As such, we cannot rule out the potential of confirmation bias, in which children may have been less likely to eat as much if they had previously reported that they were not very hungry [53]. We did, however, ask children about their level of hunger prior to both the food and non-food advertising exposures, so it is likely that any effects would have been non-differential across conditions.

Children did not compensate for their increased snack intake after food advertising during their days at the camp, however it is possible that they may have eaten less at mealtimes later in the day, or on subsequent days. Whilst we assessed children’s breakfast intake and measured their daily snack and lunch intakes we did not capture children’s food intake once they left the camp for the day. The limitations and challenges of collecting dietary intake data among free-living populations, particularly children, are well documented; they pose a high respondent burden, prompt an alteration of usual dietary habits and there is a poor accuracy when reporting food eaten away from home [54]. As such, we would not have been able to maintain the precision of the dietary intake data we had collected during camp hours if we had extended dietary data collection outside this controlled environment. There remain questions over whether children would have compensated for their increased energy intake at later eating occasions. As previously discussed, self-regulation tends to lessen over the course of the day [45]. Additionally, although research with young children (3 − 6 years) suggests that they adjust their daily food intake according to the energy density of their diets [55], studies with older children indicate that this ability to accurately compensate tends to decrease with age [56, 57], and is weaker in children with heavier weight status [58]. Whilst camp leaders ensured children’s camp activities were of a similar intensity and duration each day, children may have compensated for their additional energy intake after food advertising via increased energy expenditure outside the camp environment. In the absence of physical activity accelerometry data we are unable to determine this.

## Conclusion

Children’s exposure to unhealthy food marketing is directly associated with an imbalance in energy intake which was not compensated for during children’s time at camp. Whilst this energy imbalance may have been compensated for at a subsequent time, it is of a magnitude that, over time, could drive a positive energy gap capable of underpinning excess weight gain in children. This energy gap is higher for children with overweight and obesity, and after exposure to TV food advertising and online advergames. These findings should inform policy specifications, including the need to focus regulatory restrictions across media platforms, and particularly online media, and that behavioural weight management interventions should address heavier children’s vulnerability to food promotions.

## Abbreviations

g: Gram
kJ: Kilojoule
NSW: New South Wales
TV: Television
